# Vitamin D Deficiency Increases the Risk of Gestational Diabetes Mellitus: A Meta-Analysis of Observational Studies

**DOI:** 10.3390/nu7105398

**Published:** 2015-10-01

**Authors:** Meng-Xi Zhang, Guo-Tao Pan, Jian-Fen Guo, Bing-Yan Li, Li-Qiang Qin, Zeng-Li Zhang

**Affiliations:** 1 Department of Labor Hygiene and Environmental Health, School of Public Health of Soochow University, 199 Renai Road, Suzhou 215123, China; zhangmengxi_suda@hotmail.com (M.-X.Z.); panguotao_suda@hotmail.com (G.-T.P.); justxiangwang@hotmail.com (J.-F.G.); 2 Department of Nutrition and Food Hygiene, School of Public Health, Soochow University, 199 Renai Road, Suzhou 215123, China; bingyanli@suda.edu.cn (B.-Y.L.); qinliqiang@suda.edu.cn (L.-Q.Q.)

**Keywords:** vitamin D deficiency, gestational diabetes mellitus, pregnancy, meta-analysis

## Abstract

The results investigating the relationship between vitamin D levels and gestational diabetes mellitus (GDM) are inconsistent. Thus, we focused on evaluating the association of vitamin D deficiency with GDM by conducting a meta-analysis of observed studies. A systematic literature search was conducted via PubMed, MEDLINE, and Cochrane library to identify eligible studies before August 2015. The meta-analysis of 20 studies including 9209 participants showed that women with vitamin D deficiency experienced a significantly increased risk for developing GDM (odds ratio (OR) = 1.53; 95% confidence intervals (CI), 1.33, 1.75) with a little heterogeneity (*I*^2^ = 16.20%, *p* = 0.252). A noteworthy decrease of 4.93 nmol/L (95% CI, −6.73, −3.14) in serum 25(OH)D was demonstrated in the participants with GDM, and moderate heterogeneity was observed (*I*^2^ = 61.40%, *p* = 0.001). Subgroup analysis with study design showed that there were obvious heterogeneities in nested case–control studies (*I*^2^ > 52.5%, *p* < 0.07). Sensitivity analysis showed that exclusion of any single study did not materially alter the overall combined effect. In summary, the evidence from this meta-analysis indicates a consistent association between vitamin D deficiency and an increased risk of GDM. However, well-designed randomized controlled trials are needed to elicit the clear effect of vitamin D supplementation on prevention of GDM.

## 1. Introduction

The prevalence of gestational diabetes mellitus (GDM) is increasing globally. It has been estimated that the total prevalence of GDM reaches almost 15%–20% [1]. Moreover, women with unmanaged gestational diabetes are at increased risk of developing type 2 diabetes mellitus (T2DM) after pregnancy; their offspring are prone to developing childhood obesity and T2DM later in life [2]. The well-established risk factors for GDM include high maternal age, obesity or maternal overweight status, prior history of GDM, family history of T2DM and so on [2,3]. However, it is not clear whether a poor vitamin D status is associated with the risk of GDM.

The non-classical functions of vitamin D are gaining attention for its closer associations between vitamin D deficiency and T2DM, heart disease, autoimmune diseases and certain types of cancers [4,5]. Vitamin D deficiency has been historically defined and recently recommended by the Institute of Medicine (IOM) as a 25(OH)D of less than 50 nmol/L. Vitamin D insufficiency has been defined as a 25(OH)D of 50–75 nmol/L [6]. In accordance with these definitions, even middle-age adults are at equally high risk with elder and children for vitamin D deficiency and insufficiency worldwide [6]. Furthermore, pregnant and lactating women who take a prenatal vitamin and a calcium supplement with vitamin D remain at high risk for vitamin D deficiency. It has been reported that 40%–100% of vitamin D deficiency during pregnancy are found in Sweden, Oman, UK, USA, Australia, Pakista, urban India, Japan and China [7,8,9,10,11].

The scientific evidence linking vitamin D deficiency with diabetes is growing large, but data investigating the relationship between vitamin D levels and GDM are inconsistent. The role of vitamin D on GDM development has been studied, and vitamin D deficiency appears to be associated with altered glucose homeostasis during pregnancy [12,13]. Interestingly, a decrease in glucose and increase in insulin levels was noted after 1,25-dihydroxyvitamin D (1,25(OH)_2_D) supplementation [14]. Contradictorily, some studies suggested that no significant differences in vitamin D status were found between women with GDM and normal glucose tolerance [15,16]. Therefore, we conducted this meta-analysis to present the effect of maternal vitamin D level on GDM.

## 2. Method

### 2.1. Data Sources

We performed a search on the PubMed, MEDLINE and the Cochrane Library up to August 2015 for relevant articles in the report of this meta-analysis. The following keywords were used in the search: “vitamin D” or “cholecalciferol” or “25-hydroxyvitamin D” or “25(OH)D” in combination with “gestational diabetes”. All eligible original studies, review articles, and other relevant studies were searched manually. We performed a meta-analysis following a predetermined protocol in accordance with the MOOSE guidelines [17]. Two authors (MX-Z and GT-P) independently scanned titles and abstracts of the identified studies.

### 2.2. Study Selection

Original research evaluating the associations between vitamin D status and pregnancy outcomes were scrutinized and subsequently selected if they fulfilled the following inclusion criteria: a. study population was pregnant women without pre-existing chronic disease; b. GDM as outcome and the control were women with normal glucose tolerance (NGT); c. contained relevant data to calculate the effect size and corresponding 95% confidence intervals (CI); d. met the predefined methodological quality assessment criteria for observational studies (supplementary data Box 1) [18]; e. only studies published in English were considered.

The quality of study was evaluated using the assessment criteria for observational studies adapted from Duckitt and Harrington [18], using participant selection, comparability of groups at baseline, and how the diagnosis of gestational diabetes was made and according to what definition. We excluded any study with a score of zero in any of the six items or a total score < 7 out of 10 maximal points.

The following data were extracted from the study reports: the first author’s last name, year of publication, country of origin, study design, number of participants, diagnosis criteria of GDM, assay method of 25(OH)D and the potential confounding variables in the adjustments.

### 2.3. Statistical Analysis

The odds ratio (OR) and weighted mean difference (WMD) were used as measures of associations between 25(OH)D levels and GDM. If the OR and 95% confidence intervals (CI) were not available for meta-analysis, these data were calculated by a 2 × 2 table using the number of vitamin D deficiency cases in the GDM and control group compared with the total number of participants in both groups. The mean and standard deviation of 25(OH)D level for each study, if not provided, was converted with median and interquartile range [19].

Forest plots were used to visually assess pooled estimates and corresponding 95% CIs. The homogeneity across studies was tested using Cochran’s Q-test at *p* < 0.1, and quantified by the *I*^2^ statistic, which represents the percentage of heterogeneity that can be attributed to the variation across studies [20]. In the presence of significant heterogeneity, a random-effects model was used to calculate the pooled effect size; otherwise, a fixed-effects model was applied [21]. We further made a sensitivity analysis to investigate the influence of a single study on the overall risk estimate by omitting one study in each turn. We also conducted subgroup analysis to explore possible explanations for heterogeneity. Potent publication bias was assessed using Begg’s test and Egger’s test and examining funnel plots. Two-tailed *p* < 0.05 was considered statistically significant. All of the data were analyzed by STATA version 11.0 (StataCorp LP, College Station, TX, USA).

## 3. Results

### 3.1. Selected Articles

**Figure 1:**
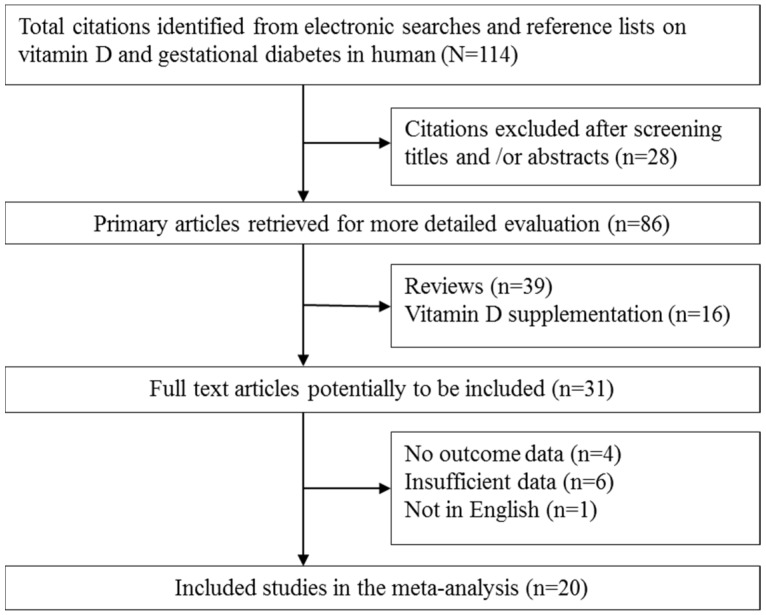
Flow chart of literature search and study selection.

The relevant 114 articles were identified in our initial search, and 82 full papers were read after screening the abstracts. Of those, 39 reviews and 16 studies aiming at vitamin D supplementation were excluded. In addition, we excluded four studies for no outcome data, six studies for insufficient data and 1 study not in English. In total, 20 studies were selected for the final analysis [15,16,22,23,24,25,26,27,28,29,30,31,32,33,34,35,36,37,38,39]. A flow chart showing the selection process is summarized in Figure 1. The quality assessment showed the quality scores ranged from 7.5 to 10, indicating that all studies were of high quality (supplementary Table S1).

### 3.2. Description of the Studies

The characteristics of the eligible studies are summarized in Table 1. These studies were published from 2008 to 2015, in which eight were conducted in Asia, seven were conducted in North America, four were conducted in Europe, and one was conducted in Australia. Eight studies had cross-sectional, five case-control, five nested case-control and two prospective cohort design. Seven different criteria were used to diagnose GDM and five assay techniques were used to measure serum 25(OH)D level.

**Table 1:** Characteristics of observational studies included in this meta-analysis.

Author and Year	Location	Study Type	Participants (*n*)	GDM (*n*)	GDM Criteria *	Assay Method	Mean 25(OH)D nmol/L (SD)		Prevalence	Significant	Adjustments**
							GDM	NGT			
Maghbooli (2008) [22]	Iran	Cross-sectional	579	52	C&C	RIA	16.5(10.4)	22.9(18.3)	70.60%	Yes	a, b
Clifton-Bligh (2008) [23]	Australia	Cross-sectional	307	81	ADPS	LC–MS	48.6(24.9)	55.3(23.3)	48%	Yes	a, b, c
Zhang (2008) [24]	US	Nested-case-control	171	57	ADA	ELISA	60.4(21.22)	75.13(24.21)	19.80%	Yes	a, b, c, d
Farrant (2009) [16]	India	Cross-sectional	559	39	C&C	RIA	49.3(31.2)	46.4(30.9)	66%	No	a, b, e, f
Soheilykhah (2010) [25]	Iran	Case-control	165	54	C&C	ELISA	24.01(20.62)	32.2(35.74)	78.40%	Yes	NR
Baker (2012) [15]	US	Nested-case-control	180	60	NDDG	LC–MS	97.0(29.0)	86.0(22.0)	7.20%	Yes	a, b, e, h
Makgoba (2011) [26]	UK	Case-control	248	90	WHO	LC–MS	47.2(26.7)	47.6 (26.7)	58.80%	No	a, b, c, d, e, g
Parlea (2012) [27]	Canada	Nested-case-control	337	118	NDDG	CLIA	56.3(19.4)	62.0(21.6)	NR	Yes	h, i
Fernandez-Alonso (2012) [28]	Spain	Cross-sectional	466	36	ADA	ECLIA	NR	NR	23.40%	NR	NR
Parildar (2013) [29]	Turkey	Case-control	122	44	IADPSG	CLIA	48.67(23.21)	57.16(24.96)	43.40%	No	NR
Wang (2012) [30]	China	Nested-case-control	400	200	ADA	ELISA	22.4(10.7)	25.9(12.3)	96.25%	Yes	a, d, j
Burris (2012) [31]	US	Cross-sectional	1155	68	ADA	CLIA	NR	NR	33.10%	NR	a, b, c, e, h, k, l, m, n, o, p, t
Perez-Ferre (2012) [32]	Spain	Cross-sectional	266	49	ADA	CLIA	NR	NR	59.02%	NR	a, c, d, g
Zuhur (2013) [33]	Turkey	Cross-sectional	402	234	IADPSG	ECLIA	30.8(16.3)	36.0(16.2)	84.30%	Yes	a, b, d, g
Bener (2013) [34]	Qatari	Prospective cohort	1873	260	WHO	RIA	NR	NR	48.40%	NR	NR
Lacroix (2014) [35]	Canada	Cross-sectional	655	54	IADPSG	LC–MS	57.5(17.2)	63.5(18.9)	26.70%	Yes	a, c, d, e, g, r, s, t, u
McManus (2014) [36]	Canada	Case-control	73	36	CDA	RIA	77.3(24.3)	93.2(19.2)	6.85%	Yes	a, b
Park (2014) [37]	Korea	Prospective cohort	523	23	C&C	ECLIA	49.4(19.4)	48(24.8)	88.90%	No	a, b, e, h, g, v
Arnold (2015) [38]	US	Nested-case-control	652	135	ADA	LC–MS	59.7(23.5)	66.6(22)	25.61%	Yes	a, b, c, d, e
Pleskacova (2015) [39]	Czech	Case-control	76	47	WHO	EIASA	28(3.76)	31.85(4.62)	94.7%	No	b

Note: NR, not reported; Prevalence: prevalence of vitamin D deficiency; Significant: significant difference in serum 25(OH)D between gestational diabetes mellitus (GDM) & normal glucose tolerance (NGT). *, Diagnostic criteria of GDM (1) C&C: Carpenter and Coustan; (2) ADPS: Australasian Diabetes in Pregnancy Society; (3) ADA: American Diabetes Association; (4) NDDG: National Diabetes Data Group; (5) WHO: World Health Organization; (6) IADPSG: International Association of the Diabetes and Pregnancy Study Groups; (7) CDA: Canadian Diabetes Association. Assay method of 25(OH)D (1) RIA: radioimmunoassay; (2) LC–MS: liquid chromatography-tandem mass spectrometry; (3) ECLIA: electrochemiluminescence immunoassay; (4) ELISA: enzyme-linked immunosorbent assay; (5) CLIA: chemiluminescence immunoassay. **, Adjustments a: age; b: body mass index (BMI); c: ethnicity; d: family history of type 2 diabetes mellitus (T2DM); e: season; f: socio-economic status; g: previous history of GDM; h: gestational age; i: maternal weight; j: triglyceride (TG); k: education; l: marital status; m: smoking; n: pregnancy weight gain; o: physical activity; p: dietary intake of fish and calcium; q: trimester; r: vitamin D lifestyle score; s: parathyroid hormone (PTH); t: parity; u: waist circumference; v: vitamin D intake.

The diversity of participant characteristics was considerable in these studies. A total of 9209 participants of whom 1737 (18.86%) were diagnosed with GDM were included with consisted of various ethnicities. Average pregnant age ranged from 24 to 35 years and the mean BMI, if provided by studies, ranged from 21.90 to 31.00 kg/m^2^. Except four studies [25,28,29,34], other research was conducted in adjustments of age, BMI, ethnicity, and family history of T2DM, *et al.* in different combinations. Prevalence of vitamin D deficiency varied considerably from 3.1% to 94.70%, and 25(OH)D level of GDM ranged from 16.50 to 97.00 nmol/L with a median of 49.30 nmol/L.

### 3.3. Main Analysis

**Figure 2:**
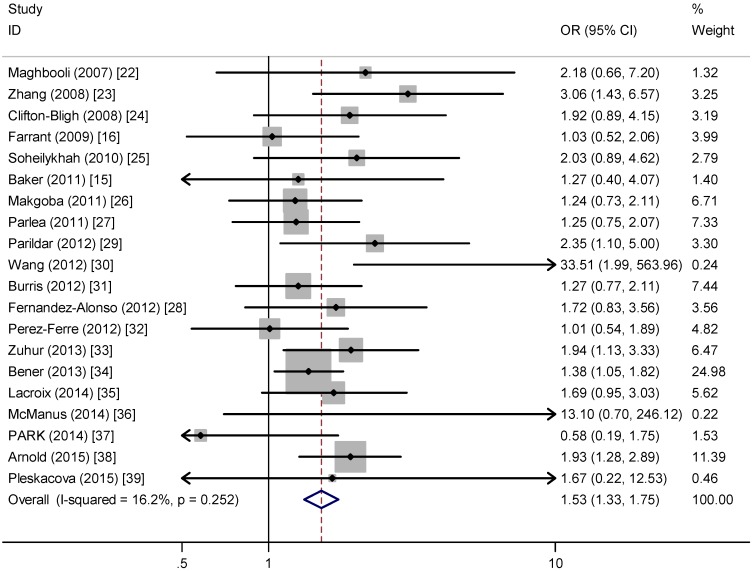
Meta-analysis of the association between vitamin D deficiency and risk of gestational diabetes mellitus (GDM).

Among the 20 studies, only six showed a significant association between vitamin D deficiency (25(OH)D levels below 50 nmol/L) and risk of GDM. However, this meta-analysis showed that women with vitamin D deficiency compared to the control group experienced a significantly increased risk for developing GDM (OR = 1.53; 95% CI, 1.33, 1.75) on a fixed-effects model (Figure 2). There was little heterogeneity across studies (*I*^2^ = 16.20%, *p* = 0.252). It is worth noting that two pooled ORs for vitamin D severe deficiency and insufficiency were calculated with the outcomes of 1.59 (95% CI, 1.11, 2.27) and 1.39 (95% CI, 1.07, 1.82), respectively (supplementary Table S2). These results indicated that vitamin D deficiency significantly increase the risk of GDM, and the higher degree of deficiency showed higher trend of GDM risk relevance.

**Figure 3:**
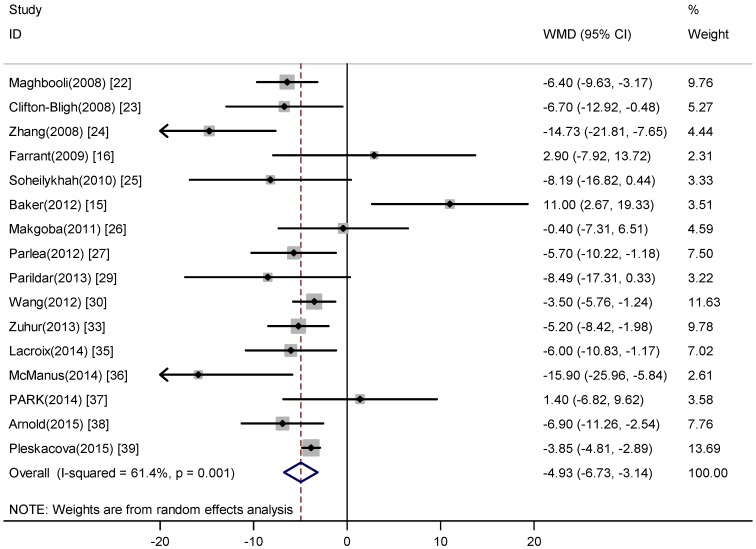
Meta-analysis of the association between serum 25(OH)D level and GDM.

Figure 3 showed the results from the random-effects model comparing the mean difference of 16 studies including 5449 participants. Ten of the sixteen studies reported a reduced level of 25(OH)D was associated with the of GDM. However, the weighted mean difference for the association varied from −15.90 to 11.00 across studies. The pooled effect was −4.93 nmol/L (95% CI, −6.73, −3.14) and significant heterogeneity was observed (*I*^2^ = 61.40%, *p* = 0.001) (Figure 3). This result showed serum 25(OH)D level was significant lower in participants with GDM than the control and also demonstrated that vitamin D deficiency is significantly associated with an increased risk of GMD.

### 3.4. Sensitivity and Subgroup Analyses

We then conducted sensitivity analyses to explore potential sources of heterogeneity in the association between weighted mean difference of 25(OH)D level and GMD, to examine the influence of various exclusion criteria on the overall risk estimate. Exclusion of two studies [15,36] in which pregnant women were vitamin D sufficient (with 25(OH)D level greater than 75 nmol/L) yielded similar results (WMD = −5.04; 95% CI, −6.43 to −3.64), and little evidence of heterogeneity was observed among the remaining studies (*I*^2^ = 37.50%, *p* = 0.077). Further exclusion of any single study did not materially alter the overall combined relative risk, with a range from −4.98 nmol/L (−6.15, −3.81) to −4.45 nmol/L (−5.50, −3.39), and each outcome were statistically significant.

Subgroup analysis with study design showed that there are obvious heterogeneities in nested case-control studies for pooled both OR and WMD (*I*^2^ = 52.5%, *p* = 0.07 for OR; *I***^2^** = 83%, *p* < 0.001 for WMD) (supplementary Figures S1 and S2). We did not perform subgroup analysis according to BMI and skin tone because of insufficient data in some studies.

### 3.5. Publication Bias

No sign of publication bias was observed when funnel plots were examined (supplementary Figure S3). The results of Begg’s (*p* = 0.163 for OR, *p* = 0.558 for WMD) and Egger’s (*p* = 0.11 for OR, *p* = 0.489 for WMD) tests also didn’t indicate evidence of publication bias.

## 4. Discussion

There has been a rapidly growing interest in the association between vitamin D and risk of gestational diabetes mellitus. The meta-analysis made in 2012 indicated a significant inverse relation of serum 25(OH)D and the prevalence of GDM from seven observational studies [40]. Similarly, our meta-analysis of 20 independent observational studies provided strong evidence that vitamin D deficiency was associated with an increased risk of gestational diabetes. Women with gestational diabetes, compared with the control group, decreased the level of 25(OH)D by 4.93 nmol/L. Vitamin D deficiency was also likely to be an independent risk factor of gestational diabetes mellitus, the same as maternal age, race, family history of diabetes, and pre-pregnancy BMI. There are several strengths of this meta-analysis compared with Poel’s study in 2012 [40]. The qualities of the included studies were relatively high. In addition, we conducted sensitivity and subgroup analyses to find which should be responsible for the heterogeneity.

We observed moderate heterogeneity among weighted mean difference as the association of GDM (Figure 3), which was not surprising given the different characteristics of participants and adjustments for confounding factors. Our sensitivity analyses suggested two studies [15,36] conducted in women with vitamin D sufficiency probably contributed to the heterogeneity. In addition to difference in features of study populations, these two studies also differed from others in some aspects. For one study, the majority of study participants were reported consuming prenatal multivitamins or taking nutritional supplements, as well as living in a geographical location which may affect the vitamin D status [15]. For the other one, the study was paused during the winter months to minimize sunlight influences, and women were also excluded if they kept all limbs modestly clothed [36]. Undoubtedly, the 25(OH)D level in two studies was high, whereas the prevalence of vitamin D deficiency was low.

Pregnancy is a state in which the mother undergoes physiological insulin resistance, which helps the fetus absorb more nutrients. Our findings for increased risk of gestational diabetes with vitamin D deficiency are biologically plausible. First, 1,25(OH)_2_D_3_, the active form of vitamin D, regulates circulating glucose levels by binding to vitamin D receptor of pancreatic β-cell and modulating insulin secretion [41,42]. Second, 1,25(OH)_2_D_3_ promotes insulin sensitivity by stimulating the expression of insulin receptors and enhancing insulin responsiveness for glucose transport [43]. Last, 1,25(OH)_2_D_3_ regulates the balance between the extracellular and intracellular calcium pools in β-cell, which is essential for insulin-mediated intracellular processes in insulin-responsive tissues [44]. In words, there is a connection between glucose metabolism and vitamin D pathways, even if further studies based on larger population are needed to get more evidence about the topic.

There are major causes behind vitamin D deficiency during pregnancy, such as inadequate exposure to sunlight, low vitamin D intake and more requirements for vitamin D. Vitamin D supplementation during pregnancy has beneficial effects on glycaemia, insulin sensitivity [45], insulin resistance [46] and metabolic profiles [47]. However, there is an active debate on the appropriate intake of vitamin D in pregnancy [12]. Apart from diet and supplements, another way of keeping optimal level of body vitamin D is from skin exposure to sunlight, which is remarkably influenced by geographical location, season, dark skin tone, sunscreen use and even the air pollution [48]. Besides, earlier studies have identified a number of factors that influence serum levels of 25(OH)D, including obesity, fat malabsorption syndrome which is unable to absorb the fat-soluble vitamin D, medications enhancing the catabolism of 25(OH)D and 1,25(OH)_2_D_3_, and so on [5].

Several limitations of this meta-analysis should be acknowledged. Firstly, the median and quartiles in some studies [16,25,30,39] were converted into mean and their SDs for meta-analysis. The calculation for skewed distribution virtually reduced the precision. Secondly, there are four types of observed study in this meta-analysis, which may result in the heterogeneity. Indeed, subgroup analysis with study design demonstrated that there are obvious heterogeneities in nested case–control studies for pooled both OR and WMD. Interestingly, we just found that two researches [23,34] of pregnant women with vitamin D sufficiency are nested case-control and case-control studies, respectively, which tests the hypothesis mentioned above better. Thirdly, there is one more point that the observed associations between maternal vitamin D status and the risk of adverse pregnancy outcomes could be affected by confounding factors, such as BMI and skin tone. Taking BMI for example, increased BMI in pregnancy is associated with higher risk of GDM [49], as well as increased risk of vitamin D deficiency [5]. Some but not all of the individual studies generated adjusted ORs, so we could not pool the findings by adjusting confounding factors. However, the association between vitamin D deficiency and GDM still remains (OR = 1.67; 95% CI, 1.31, 2.13; *I*^2^ = 25.6%, *p* = 0.251) even after adjusting for maternal age, BMI and ethnicity (supplementary Figure S4). Finally, different diagnostic criteria of GDM could have influenced the pooled effect due to different threshold value for oral glucose tolerance test.

## 5. Conclusions

In conclusion, the evidence from this article indicates a consistent association between vitamin D deficiency and an increased risk of gestational diabetes mellitus. A significant decrease of 4.93 nmol/L in serum 25(OH)D was demonstrated in the participants with GDM. However, well-designed randomized controlled trials are needed to determine the explicit effect of vitamin D supplementation on prevention of GDM. Until then, screening women who are at risk of vitamin D deficiency and supplementation with vitamin D could be considered.

## Supplementary Materials

Supplementary materials can be found at: http://www.mdpi.com/2072-6643/7/10/5398/s1.
