# Supplementary material for: Vitamin D Deficiency Increases the Risk of Gestational Diabetes Mellitus: A Meta-Analysis of Observational Studies
Source: Nutrients. 2015 Oct 1;7(10):8366–75. doi: 10.3390/nu7105398 (PMC4632418; doi:10.3390/nu7105398)
Supplement: Supplementary file 1 [file nutrients-07-05398-s001.docx]

Supplementary Information

**Box 1.** Quality assessment of observational studies (total 10 points)*

**1. Selection of participants (1/0)**

*Cohort studies (1/0)*

Selected cohort was representative of the general population (population-based studies) or target catchment population (hospital-based studies) (1)

Cohort was a selected unrepresentative group (0)

*Case control studies (1/0)*

Cases and controls drawn from the same population (1)

Cases and controls drawn from different sources or the selection of groups (0)

**2. Comparability of groups (2/0)**

No significant differences between the groups reported in terms of age, plurality, smoking, history of preterm birth, preeclampsia or gestational diabetes, pre-existing medical conditions were explicitly reported, or these differences were adjusted for (2)

Differences between groups were not examined (1)

Groups differed and no adjustment results provided (0)

**3. Definition of outcomes (2/0)**

Definition of outcomes

Referenced or standard definition (2)

Explicit non-standard definition (1)

Unspecified or unacceptable definition (0)

**4. Ascertainment of outcomes (2/0)**

How the diagnosis was made

Prospectively diagnosed or review of notes/hospital discharge records (2)

Retrospective chart review or database coding (1)

Process not described (0)

**5. Sample size (1/0)**

≥200 participants in a cohort study; ≥50 participants in either group (case/control) (1)

100≤ participants <200 in a cohort; 25≤ participants <50 in either group (case/control) (0.5)

Participants <100 or total number of events <10 in a cohort; participants <25 in either group (case/control) (0)

**6. Study design (2/0)**

Prospective cohort or nested case-control within a prospective cohort (2)

Cross-sectional, case-control or retrospective cohort (1)

Not described or poorly designed (0)

**Exclusion:** score zero in any item (1 to 6) or a total score <7 out of 10 maximal points

*A score based quality assessment criteria for non-randomized observational studies adapted from Duckitt & Harrington [[26](#_ENREF_26)]

**Table S1.** Quality scores of included studies on vitamin D status and pregnancy outcomes.

| **Study** | **Selection of Participants** | **Comparability of Groups** | **Outcomes Definition** | **Ascertainment** | **Sample Size** | **Study Design** | **Total Score** |
| --- | --- | --- | --- | --- | --- | --- | --- |
|  |  |  |  |  |  |  |  |
| Maghbooli (2007) [[22](#_ENREF_20)] | 1 | 2 | 2 | 2 | 1 | 1 | 9 |
| Clifton-Bligh (2008) [[23](#_ENREF_21)] | 1 | 2 | 2 | 2 | 1 | 1 | 9 |
| Zhang (2008) [[24](#_ENREF_22)] | 1 | 2 | 2 | 2 | 1 | 2 | 10 |
| Farrant (2009) [16] | 1 | 1 | 2 | 2 | 1 | 1 | 8 |
| Soheilykhah (2010) [2[5](#_ENREF_24)] | 1 | 1 | 2 | 2 | 1 | 1 | 8 |
| Baker (2011) [[15](#_ENREF_25)] | 1 | 2 | 2 | 2 | 1 | 2 | 10 |
| Makgoba (2011) [[26](#_ENREF_26)] | 1 | 1 | 2 | 2 | 1 | 1 | 8 |
| Parlea (2011) [[27](#_ENREF_27)] | 1 | 2 | 2 | 2 | 1 | 2 | 10 |
| Fernandez-Alonso (2011) [[28](#_ENREF_28)] | 1 | 1 | 2 | 2 | 1 | 1 | 8 |
| Parildar (2012) [[29](#_ENREF_29)] | 1 | 1 | 2 | 2 | 0.5 | 1 | 7.5 |
| Wang (2012) [[30](#_ENREF_30)] | 1 | 2 | 2 | 2 | 1 | 2 | 10 |
| Burris (2012) [[31](#_ENREF_31)] | 1 | 2 | 2 | 2 | 1 | 1 | 9 |
| Perez-Ferre (2012) [[32](#_ENREF_32)] | 1 | 2 | 1 | 2 | 1 | 1 | 8 |
| Zuhur (2013) [[33](#_ENREF_33)] | 1 | 2 | 2 | 2 | 1 | 1 | 9 |
| Bener (2013) [[34](#_ENREF_34)] | 1 | 2 | 2 | 2 | 1 | 2 | 10 |
| Lacroix (2014) [[35](#_ENREF_35)] | 1 | 2 | 2 | 2 | 1 | 1 | 9 |
| McManus (2014) [[36](#_ENREF_36)] | 1 | 2 | 2 | 2 | 0.5 | 1 | 8.5 |
| Park (2014) [[37](#_ENREF_37)] | 1 | 2 | 2 | 2 | 1 | 2 | 10 |
| Arnold (2015) [[38](#_ENREF_38)] | 1 | 2 | 2 | 2 | 1 | 2 | 10 |
| Pleskacova (2015) [[39](#_ENREF_39)] | 1 | 2 | 2 | 2 | 0.5 | 1 | 8.5 |

**Table S2.** Results of meta-analysis according to 25(OH)D level.

| **Vitamin D Status** | **25(OH)D nmol/L** | **GDM**  **(n)** | **NGT**  **(n)** | **OR** | **95% CI** | ***I*^2^**  **%** | **P** |
| --- | --- | --- | --- | --- | --- | --- | --- |
| severe deficiency | <25 | 338 | 970 | 1.59 | 1.11, 2.27 | 64.60 | 0.01 |
| deficiency | <50 | 1024 | 3622 | 1.53 | 1.33, 1.75 | 16.20 | 0.252 |
| insufficiency | <75 | 778 | 4646 | 1.39 | 1.07, 1.82 | 47.40 | 0.04 |

**Figure S1.** The results of subgroup analysis according to study design (OR).

**Figure S2.** The results of subgroup analysis according to study type (WMD).

|  |
| --- |
| (**A**) |
|  |
| (**B**) |

**Figure S3.** Funnel plot of the association between maternal serum 25(OH)D and GDM (**A**): OR;
(**B**): WMD).

**Figure S4.** The results of meta-analysis after adjusting for maternal age, BMI and ethnicity.

© 2015 by the authors; licensee MDPI, Basel, Switzerland. This article is an open access article distributed under the terms and conditions of the Creative Commons by Attribution (CC-BY) license (http://creativecommons.org/licenses/by/4.0/).
